# Parapharyngeal Metastasis of Papillary Carcinoma of Thyroid Gland: A Case Report and a Review of the Literature

**DOI:** 10.3390/diagnostics13081426

**Published:** 2023-04-15

**Authors:** Massimo Campagnoli, Davide Masnaghetti, Maria Silvia Rosa, Edoardo Paganelli, Massimiliano Garzaro, Paolo Aluffi Valletti

**Affiliations:** 1 ENT Department, University of Piemonte Orientale, 28100 Novara, Italy; orl@maggioreosp.novara.it (D.M.); m.silviarosa@gmail.com (M.S.R.);; 2 ENT Department, Michele e Pietro Ferrero Hospital, 12060 Verduno, Italy

**Keywords:** thyroid, parapharyngeal space, metastasis, ENT, differentiated thyroid cancer, papillary carcinoma, therapy

## Abstract

Papillary carcinoma is the most frequently encountered differentiated thyroid carcinoma. Usually, metastasis occurs along lymphatic pathways in the central compartment and along the jugular chain. Nevertheless, lymph node metastasis in the parapharyngeal space (PS) is a rare but possible event. In fact, a lymphatic pathway has been identified that connects the upper pole of the thyroid and the PS. We describe the case of a 45-year-old man with a two-month history of a right neck mass. He underwent a complete diagnostic path that highlighted the presence of a parapharyngeal mass associated with the presence of a thyroid nodule suspected to be malignant. The patient underwent surgery (thyroidectomy and removal of the PS mass that was found to be a metastatic node of papillary thyroid carcinoma). The aim of this case is to underline the importance of detecting these kinds of lesions. Nodal metastasis in PS from thyroid cancer is a rare occurrence that is not easily detectable by a clinical examination until the metastasis reaches a considerable dimension. Computed tomography (CT) and magnetic resonance imaging (MRI) permit early identification, but unfortunately, these are not usually employed as a first-level imaging technique in patients with thyroid cancer. The treatment of choice is surgery with a transcervical approach that allows for better control of the disease and of the anatomical structures. Non-surgical treatments are usually reserved for patients with advanced disease, with satisfactory results.

## 1. Introduction

Thyroid carcinoma is the most frequent endocrine neoplasm. It is estimated that about 12,200 new cases of thyroid carcinoma were diagnosed in Italy in 2019, of which three-quarters were diagnosed in the female sex. The incidence of thyroid carcinoma has progressively increased in almost all countries during the last decades. In 2019, in the Italian population under 50 years old, thyroid carcinoma was the second most frequent neoplasm in the female sex (5% of all neoplasms) and the third most frequent in men (8% of all neoplasms) [1]. Well-differentiated thyroid cancers are characterized by a 20–50% rate of regional lymph node metastases [2].

Among all of the thyroid malignancies, papillary thyroid carcinoma (PTC) is the most frequently encountered histological type, accounting for 80–85% of all thyroid cancer cases [3]. Papillary thyroid carcinoma is a malignancy showing evidence of follicular cell differentiation and a set of distinctive nuclear characteristic features. The carcinoma usually appears as an asymptomatic irregular solid mass, but in rare cases, it may have cystic features. PTC is characterized by its ability to invade the outlying tissues and lymphatics; in fact, about 10% of patients present with metastatic disease at the moment of first diagnosis. Metastasis usually occurs along the lymphatic pathways in the central compartment and along the jugular chain. The overall prognosis is good for most patients, especially those younger than 45 years of age [4]. In some sources, it is described that in approximately 20% of cases, patients complain about hoarseness and dysphagia as the first symptoms, signaling recurrent laryngeal nerve involvement with vocal cord paralysis or tracheal compression. Lymph node metastasis in the parapharyngeal space (PS) associated with PTC is a rare but possible event. A lymphatic pathway that connects the upper pole of the thyroid and the PS has been identified [5]. To the best of our knowledge, only 112 cases of metastases in the PS have been reported in literature in the last 20 years, and the reported incidence of PS metastases of well-differentiated thyroid cancers ranges from 0.43% to 2.5% [6].

The PS is an extremely complex anatomical region that presents an inverted cone shape. It extends from the base of the skull to the hyoid bone [6].

**Figure 1:**
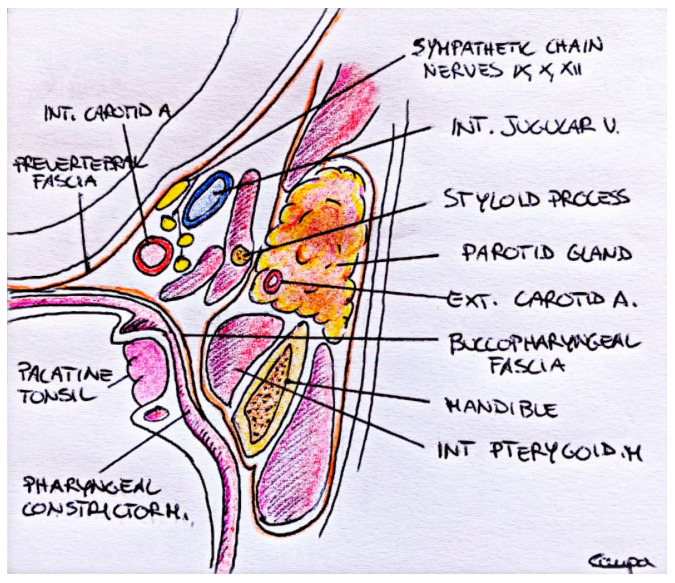
Axial section of parapharyngeal space anatomy showing fascia delimiting this space and main muscular, vascular, and nervous structures crossing PS.

The lateral limit of this region is represented by the fascia covering the inner surface of the pterygoid muscles, the posterior belly of the digastric muscle, and the deep lobe of the parotid gland. The PS is medially defined by the alar fascia and the buccopharyngeal fascia that cover the superior pharyngeal constrictor muscle and the pharyngobasilar fascia. The alar fascia is a thin and often dehiscent structure that separates the PS from the retropharyngeal space and at the same time permits the internal carotid artery to reach the midline. The posterior layer of the carotid artery sheath corresponds to the PS posterior limit [7]. The PS is divided into prestyloid and poststyloid spaces by tensor-vascular-styloid fascia. This fascia is superiorly composed of the tensor veli palatini muscle and fascia and inferiorly composed of the stylopharyngeal and styloglossus muscles (Figure 1).

In the poststyloid compartment, in addition to neural and vascular structures, multiple lymph nodes are present [8]. They might be involved in malignancies of the pharynx, sinonasal tract, and, in very rare instances, of the thyroid gland.

Metastasis of this compartment sometimes appears as a solitary asymptomatic laterocervical node. Every time an adult patient presents with a silent neck mass, it should be considered malignant until proven otherwise. For this reason, an accurate anamnesis should be performed. A clinical evaluation should also be performed, including an endoscopic evaluation of the upper airways. On the basis of the results, a diagnostic path should be set up, including blood tests, imaging (ultrasound, CT, MRI, or positron emission tomography), and biopsy (open or fine-needle), with the aim of reaching a diagnosis as soon as possible and proposing the best treatment option to the patient.

We report a case of papillary thyroid carcinoma with the concomitant presence of a PS metastasis.

## 2. Case

A 45-year-old man with a 2-month history of a painless right neck mass was referred to our clinic. He related that he noticed the mass while shaving his beard, and during the time since then, it grew slowly. He consulted with his family doctor several times, who suggested antibiotic and steroid therapy with no benefit. The patient had no dysphonia, dysphagia, dyspnea, or reflex otalgia. No significant ponderal loss was reported. The patient had no history of thyroid or head and neck disease, and no other comorbidities were reported. During the clinical examination, a cervical mass of about 3.5 cm was observed in the right cervical region. It was hard at palpation, not easily mobile in relation to deep and superficial tissues, and the overlying skin was unaffected. At thyroid palpation, a right hard nodule was noted in the right lobe with a gross diameter of 4 cm.

No other masses were present during palpation of the neck. No oral lesions were present. The oropharynx did not present considerable findings. An endoscopy did not show laryngeal, pharyngeal, or hypopharyngeal lesions; laryngeal motility was present, and no salivary stagnation was evident. In order to complete the diagnostic workup, the patient underwent a neck ultrasound (US) and fine-needle biopsy (FNAC) of the thyroid nodule. The neck US evidenced a 4.2 cm thyroid nodule with necrosis in the center and multiple calcification spots. No lymph nodes of the jugular chain were evident. Fine-needle aspiration was performed both on the thyroid and cervical nodules; cytological examination detected the presence of a cellular population, suggesting the presence of a papillary thyroid carcinoma on the thyroid node (Thy5). The parapharyngeal mass was characterized as a plausible metastasis of papillary carcinoma.

In order to complete the study of the PS mass, computed tomography with contrast and magnetic resonance with contrast were performed.

**Figure 2:**
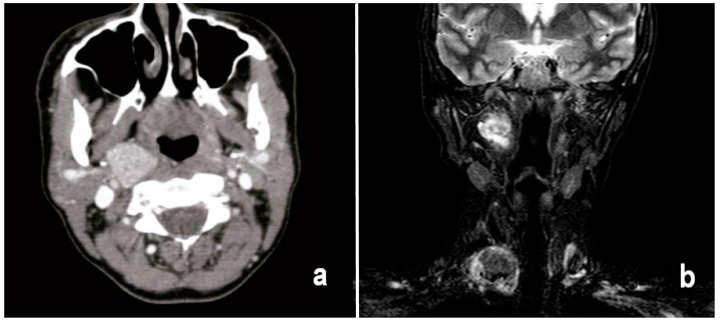
(**a**) Axial CT scan of the parapharyngeal node; (**b**) coronal MRI scan showing thyroid mass and parapharyngeal metastatic node.

Both computed tomography and magnetic resonance imaging revealed the presence of a right thyroid node with a diameter of 4.5 × 3.8 cm characterized by a central necrotic–hemorrhagic nucleus, as well as the presence of a right parapharyngeal mass with a diameter of 3.8 × 3 cm (Figure 2).

Serological examination showed normal levels of fT3, fT4, TSH, and calcitonin.

Considering the characteristics of the disease, as well as the good medical condition and young age of the patient, a surgical approach was proposed to the patient.

Informed consent to the surgical procedure was received from the patient.

The patient underwent a total thyroidectomy, a level VI lymph node dissection, a right modified radical neck dissection, and asportation of the parapharyngeal mass.

**Figure 3:**
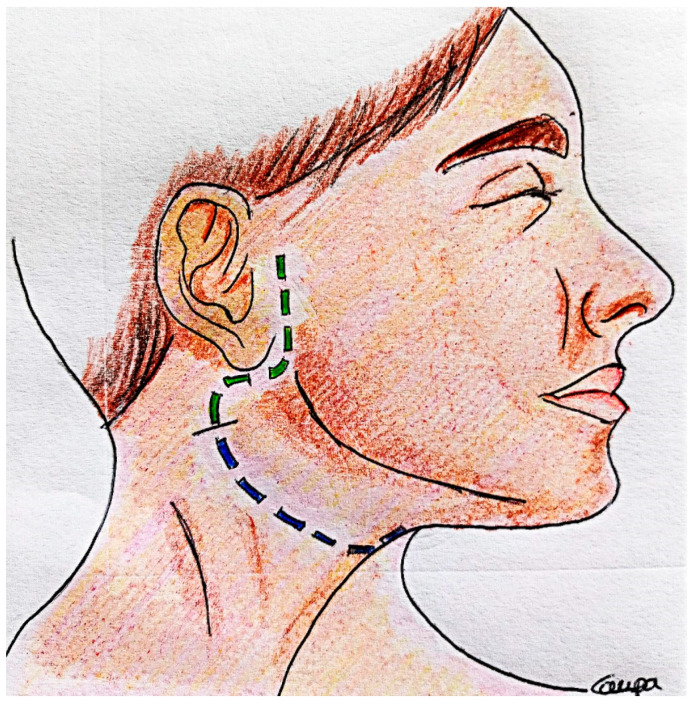
Skin incision for transparothid (green) and cervical (blue) approaches to PS.

**Figure 4:**
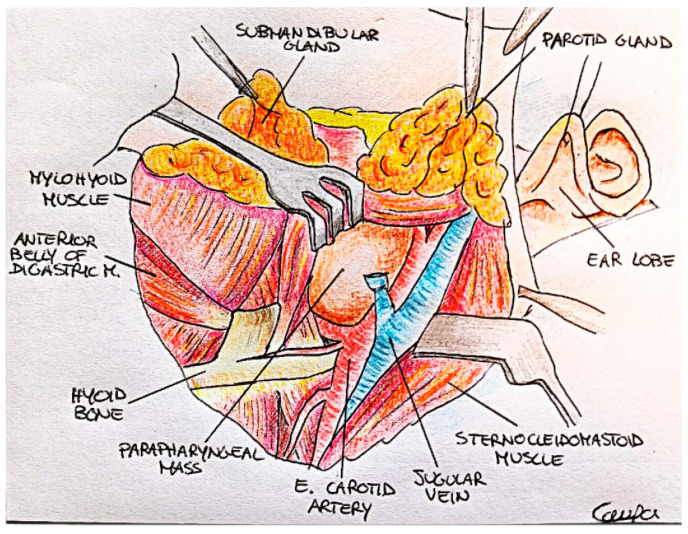
Representation of the operating field. It is evident that by elevating the posterior belly of the digastric muscle (up left retractor) and retracting the sternocleidomastoid muscle (inferior right Farabeuf retractor), it is possible to identify the vascular cervical axis and gain the access to PS.

Asportation of the parapharyngeal mass, located in the prestyloid region, was performed with a transcervical approach with a section of the digastric and stylohyoid muscles (Figure 3 and Figure 4).

The postoperative course was uneventful. Surgical drainage of the thyroid region was removed 48 h after surgery; surgical drainage of the parapharyngeal and laterocervical regions was removed after a further 24 h. Oral feeding was permitted on the second postoperative day. The patient did not complain of dysphonia. An endoscopic evaluation was performed the day after surgery, before allowing oral feeding and before discharge; no laryngeal motility impairment, salivary stagnations, or pharyngeal–laryngeal swallowing were evident. The patient was discharged from the hospital 4 days after surgery.

Histological examination of the operative specimens confirmed the presence of a papillary thyroid carcinoma of the right thyroid lobe and revealed the presence of a lymph nodal metastasis of the right VI level and of the parapharyngeal node. No metastasis was found in the laterocervical lymph nodes.

The tumor was classified as being stage pT3 N1b.

After the surgical treatment, the patient underwent an endocrinological evaluation with an indication for a radioactive iodine treatment and received the normal oncological follow-up.

After 5 years of follow-up, the patient was disease free.

## 3. Discussion

The parapharyngeal space (PS) could be occupied by four different types of neoplastic lesions: a primary tumor of the PS, lymph node involvement by lymphoproliferative disease, tumors arising in adjacent sites, and the involvement of the PS with metastatic lymph nodes.

Neoplasms involving the PS are a rare event; in fact, they represent 0.5% of all head and neck neoplasms [9].

Post-styloid lymph nodes are usually involved in parapharyngeal or sinonasal squamous cell carcinoma and lymphomas.

The presence of metastatic disease from papillary carcinoma of the thyroid gland, as shown in our case, is extremely rare; it is estimated that only 0.4% to 5% of patients affected by thyroid cancer have metastasis in the PS or the retropharyngeal space (RPS) [10].

Diagnosis of parapharyngeal nodal metastases could be very difficult. They commonly present as a painless neck mass or a globus sensation in the throat due to bulging of the oropharynx [11]. PS and RPS lymph nodes are hardly detectable on physical examination or nasopharyngoscopy, so radiological imaging is essential in order to find them [11,12]. In these anatomical regions, metastatic nodes can be clinically detected only when they reach a size of approximately 2.5–3.0 cm, as happened in our case [13].

In patients with thyroid cancer, US is the standard of care for preoperative imaging of the central and lateral compartments of the neck. Because of this, lymph nodes located in the PS/RPS could easily remain undetected until they reach a considerable dimension, becoming symptomatic [12]. An US was also performed in our patient, together with FNA of the lesions and blood tests, as the first diagnostic step. A CT scan and MRI were necessary to complete the diagnostic evaluation. In general, CT scans allow for the study of deep neck structures, and contrast-enhanced CT scans can facilitate the diagnosis of tumors in the poststyloid parapharyngeal space [14]. An MRI provides better tissue characterization and definition of tumor margins, whereas a CT better identifies bony details and landmarks. CT and MRI are comparable in their diagnostic yield of RP/PP space tumors [10].

Lombardi et al. tried to identify specific imaging characteristics of PS/RPS nodal metastasis from thyroid cancer. They found that in both CT and MRI, a cystic aspect of the metastatic node is present in up to 70% of cases. This might be explained by the fact that thyroid carcinomas have a tendency to produce a large amount of colloid; moreover, their high vascularization can promote the onset of intralesional hemorrhage. Punctate calcifications might be an expression of psammomatoid bodies, and they can be easily detected by ultrasonography and CT [9].

In a recent systematic review with 239 patients, it was observed that metastases of the RPS/PS are generally recurrent, and they are often associated with aggressive, high-risk disease. However, it is uncertain whether this is a true phenomenon or the result of more extensive use of imaging in the secondary setting [10].

Previous lateral neck dissection is considered a risk factor for the development of PS/RPS metastases from thyroid cancer [15]. 

Kaplan et al. concluded that RP nodal metastases may be more frequent in patients who have previously undergone treatment for thyroid cancer, whereas parapharyngeal nodal metastases are more likely to occur in untreated patients [16].

Nevertheless, Rouvier described a lymphatic drainage connecting the upper and posterior compartments of the lateral thyroid lymph vessels to the RPS lymphatic system; according to his opinion, this variation is present in 20% of patients [17].

In a review published in 2021, the results showed that 72.3% of all cases presented as a recurrence of previously treated thyroid carcinoma. In almost 50% of these patients, a neck dissection was performed. The authors found that the prevalence of RPS vs. PS metastases and the number of previous neck dissections in initial vs. recurrent patients was not significantly different [10].

Concerning the presence of aggressive histologic features in these patients, there are limited data in the literature, perhaps because the criteria for defining histological high-risk thyroid cancer (e.g., tall cell carcinoma) have changed over time; moreover, histologic features are rarely reported in the literature [10].

Surgery is typically the treatment of choice for RPS/PS tumors [11,18]. The transcervical approach should be considered the optimal choice, as it allows for a more complete removal of the mass and permits good exposure of the prestyloid and poststyloid compartments, including major vessels such as the carotid artery [9,19]. This approach is particularly useful in cases also requiring a concurrent neck dissection; however, the transcervical approach poses a risk of injury to the lower cranial nerves and cervical sympathetic chain [18]. Transoral approaches should be considered only in cases of isolated, well-encapsulated, and small nodes that do not transgress the styloid process. This approach provides limited surgical access and should therefore not be used in cases in which extranodal extension is suspected [20]. When this kind of approach is chosen, a careful imaging study is recommended to identify the relationship between the node and the vascular structures [8]. Endoscope-assisted and transoral robotic resections are additional possibilities for this procedure [21].

Other methods include endoscope-assisted, VITOM^®^ 3D-assisted, and transoral robotic resections [22].

The execution of these procedures should be performed by an experienced surgeon. In fact, the PS is traversed by many vascular and nervous noble structures that, if damaged, could leave the patient with many important sequelae that, in some cases, could be life threatening [9]. 

The most frequent and fearsome complications of the surgery are palatal insufficiency, tongue weakness, Horner’s syndrome, vocal cord paralysis, facial nerve weakness, and hemorrhage [22].

In cases in which parapharyngeal metastasis from thyroid cancer is diagnosed preoperatively, surgical treatment should include a total thyroidectomy and the removal of the parapharyngeal metastasis, whether it is associated with neck dissection or not. Because of the possible presence of a direct lymphatic drainage from the thyroid to the PS and the RPS, the presence of a metastasis in these areas may not be associated with an increased risk of developing metastasis in level II to IV. For these reasons, neck dissection should be performed only in the case of clinical/ultrasonographic suspicion of laterocervical metastasis [9,21].

The surgical management of nodal metastasis of the PS/RFS from thyroid cancer is easier in the initial setting, as opposed to the recurrent setting, where scarring complicates surgical removal.

Moreover, a complete resection of the nodal metastasis facilitates a biochemical follow-up with thyroglobulin levels.

Postoperative radioactive iodine therapy (RAI) is administered in some cases of RP/PP metastases [6], whereas primary external beam radiotherapy is generally reserved for patients with unresectable tumors, compromised airways, and/or poorly differentiated cancers [11,23].

The cases reported in the literature underwent a variety of different treatments, which did not always involve surgery; though surgery has always been considered the option with the best chance of curative treatment, this could not be proven with statistical evidence [10].

## 4. Conclusions

Nodal metastases in the RPS/PS from thyroid cancer are a rare occurrence. They are not easily detectable by a clinical examination before they reach a considerable dimension. CT and MRI permit early identification, but unfortunately, these are not usually employed as a first-level imaging technique in patients with thyroid cancer. These metastases occur more frequently in patients with recurrence and in those who underwent a previous surgical treatment for thyroid cancer. The treatment of choice is surgery with a transcervical approach that allows for better control of the disease and of the anatomical structures. A transoral approach is permitted but only in extremely selective cases. Non-surgical treatments (RAI and external beam radiotherapy) are usually reserved for patients with advanced disease, with satisfactory results.

## Data Availability

Data are available upon request to the corresponding author’s e-mail address.
